# Consensus document for the diagnosis of peripheral bone infection in adults: a joint paper by the EANM, EBJIS, and ESR (with ESCMID endorsement)

**DOI:** 10.1007/s00259-019-4262-x

**Published:** 2019-01-24

**Authors:** Andor W. J. M. Glaudemans, Paul C. Jutte, Maria Adriana Cataldo, Victor Cassar-Pullicino, Olivier Gheysens, Olivier Borens, Andrej Trampuz, Klaus Wörtler, Nicola Petrosillo, Heinz Winkler, Alberto Signore, Luca Maria Sconfienza

**Affiliations:** 1 Department of Nuclear Medicine and Molecular Imaging, Medical Imaging Center, University of Groningen, University Medical Center Groningen, Hanzeplein 1, 9700 RB Groningen, The Netherlands; 2 Department of Orthopaedic Surgery, University of Groningen, University Medical Center Groningen, Groningen, The Netherlands; 3 Clinical and Research Department on of Infectious Diseases, “L. Spallanzani”, IRCCS-Rome, Rome, Italy; 4 Department of Diagnostic Imaging, Robert Jones & Agnes Hunt Orthopaedic Hospital NHS Trust, Oswestry, Shropshire UK; 5 Department of Nuclear Medicine and Molecular Imaging, University Hospitals Leuven, Leuven, Belgium; 6 Division of Orthopaedic Surgery and Traumatology, Lausanne University Hospital, Lausanne, Switzerland; 7 Center for Muskuloskeletal Surgery, Charité - Universitätsmedicin Berlin, Berlin, Germany; 8 69 Division Institut für Diagnostische und Interventionelle Radiologie, Klinikum Rechts der Isar, Technische Universität München, Munich, Germany; 9 Osteitis-Centre, Privatklinik Döbling, Vienna, Austria; 10 Nuclear Medicine Unit, Faculty of Medicine and Psychology, Department of Medical-Surgical Sciences and Translational Medicine, “Sapienza” University, Rome, Italy; 11 Unit of Diagnostic and Interventional Radiology, IRCCS Istituto Ortopedico Galeazzi, Milan, Italy; 12 Department of Biomedical Sciences for Health, Università degli Studi di Milano, Milan, Italy

**Keywords:** Peripheral bone infection, Osteitis, Osteomyelitis, Diagnosis of infection, Guideline, Imaging

## Abstract

**Introduction:**

In adults with a suspicion of peripheral bone infection, evidence-based guidelines in choosing the most accurate diagnostic strategy are lacking.

**Aim and methods:**

To provide an evidence-based, multidisciplinary consensus document on the diagnostic management of adult patients with PBIs, we performed a systematic review of relevant infectious, microbiological, orthopedic, radiological, and nuclear medicine literature. Delegates from four European societies (European Bone and Joint Infection Society, European Society of Microbiology and Infectious Diseases, European Society or Radiology, and European Association of Nuclear Medicine) defined clinical questions to be addressed, thoroughly reviewed the literature pertinent to each of the questions, and thereby evaluated the diagnostic accuracy of each diagnostic technique. Inclusion of the papers per statement was based on a PICO (Population/problem – Intervention/indicator – Comparator – Outcome) question following the strategy reported by the Oxford Centre for Evidence-based Medicine. For each statement, the level of evidence was graded according to the 2011 review of the Oxford Centre for Evidence-based Medicine. All approved statements were addressed taking into consideration the available diagnostic procedures, patient acceptance, tolerability, complications, and costs in Europe. Finally, a commonly agreed-upon diagnostic flowchart was developed.

**Electronic supplementary material:**

The online version of this article (10.1007/s00259-019-4262-x) contains supplementary material, which is available to authorized users.

## Preamble

The European Association of Nuclear Medicine (EANM) is a professional nonprofit medical association that facilitates communication worldwide between individuals pursuing clinical and research excellence in nuclear medicine. The EANM was founded in 1985. EANM members are physicians, technologists, and scientists specializing in the research and practice of nuclear medicine.

The EANM will periodically define new guidelines for nuclear medicine practice to help advance the science of nuclear medicine and to improve the quality of service to patients throughout the world. Existing practice guidelines will be reviewed for revision or renewal, as appropriate, on their fifth anniversary or sooner, if indicated.

Each practice guideline, representing a policy statement by the EANM, has undergone a thorough consensus process in which it has been subjected to extensive review. The EANM recognizes that the safe and effective use of diagnostic nuclear medicine imaging requires specific training, skills, and techniques, as described in each document. Reproduction or modification of the published practice guideline by those entities not providing these services is not authorized.

The present guideline was developed collaboratively by the EANM with the European Society of Radiology (ESR), the European Bone and Joint Infection Society (EBJIS), and the European Society of Microbiology and Infectious Diseases (ESCMID).

These guidelines are an educational tool designed to assist practitioners in providing appropriate care for patients. They are not inflexible rules or requirements of practice and are not intended, nor should they be used, to establish a legal standard of care. For these reasons and those set forth below, the involved societies caution against the use of these guidelines in litigation in which the clinical decisions of a practitioner are called into question.

The ultimate judgment regarding the propriety of any specific procedure or course of action must be made by the physician or medical physicist in light of all the circumstances presented. Thus, there is no implication that an approach differing from the guidelines, standing alone, is below the standard of care. To the contrary, a conscientious practitioner may responsibly adopt a course of action different from that set forth in the guidelines when, in the reasonable judgment of the practitioner, such course of action is indicated by the condition of the patient, limitations of available resources, or advances in knowledge or technology subsequent to publication of the guidelines.

The practice of medicine includes both the art and the science of the prevention, diagnosis, alleviation, and treatment of disease. The variety and complexity of human conditions make it impossible to always reach the most appropriate diagnosis or to predict with certainty a particular response to treatment. Therefore, it should be recognized that adherence to these guidelines will not ensure an accurate diagnosis or a successful outcome. All that should be expected is that the practitioner will follow a reasonable course of action based on current knowledge, available resources, and the needs of the patient to deliver effective and safe medical care. The sole purpose of these guidelines is to assist practitioners in achieving this objective.

## Introduction

Peripheral bone infections (PBI) include osteitis and osteomyelitis. Osteitis is an external bacterial infection of the bone and surrounding soft tissues after trauma and/or surgery, and can be divided in acute (within the first 8 weeks) and chronic (> 8 weeks). Osteomyelitis refers to a primary infection of the bone marrow (mostly endogenous by hematogenous spread) with subsequent involvement of the cortical bone [1]. The strategy to diagnose an infection of the bone is similar for osteitis and for osteomyelitis. In the literature, osteitis and osteomyelitis are used interchangeably, no clear distinction is used between the two entities.

The most frequent origin of osteomyelitis is exogenous, following trauma or surgery, but they also can develop by contiguous spread from adjacent soft tissue or joint infection. A hematogenous spread of microorganisms may also be the origin of the infection [2]. In the elderly, a reactivation of a site of quiescent hematogenous osteomyelitis acquired during childhood can occur [3]. Identification of patients with a foreign-body implant is important, both because of their high susceptibility to infection and because of treatment challenges. People with diabetes may suffer from osteomyelitis secondary to vascular insufficiency following a foot soft tissue infection that spreads to bone [2].

Osteomyelitis can be classified as acute or chronic. The hallmark of acute osteomyelitis is the simultaneous presence of pathogens and necrotic bone. Acute osteomyelitis, if not promptly and efficaciously treated, becomes a chronic infection, which is characterized by the presence of a necrotic piece of devascularized bone, called sequestrum. A periosteal reactive process with new bone formation develops around the necrotic area and may be associated with sinus tracking to the skin surface [4]. Regarding the etiology, hematogenous osteomyelitis is almost always a monomicrobial infection. *Staphylococcus aureus* is the most frequent causative microorganism followed by coagulase-negative staphylococci, aerobic Gram-negative bacteria, and *Peptostreptococcus* spp. [5]. In contrast to hematogenous osteomyelitis, secondary osteomyelitis is more frequently polymicrobial. *Staphylococcus aureus* and coagulase-negative staphylococci account for most bacteria isolated in this type of osteomyelitis. However, Gram-negative bacilli and anaerobic organisms are also frequently isolated [3].

PBI behave differently from infections of the axial skeleton, especially spine, and therefore diagnostic possibilities differ between infections in peripheral bone compared to axial bone. The etiology, behavior, and diagnostic possibilities also differ between children (e.g., more acute osteomyelitis) and adults. This guideline focuses exclusively on PBI in adults. Infections in the diabetic foot are excluded in this guideline, since these infections behave differently due to vascular and neuropathic impairment. Guidelines for the diagnosis of diabetic foot infection are being developed in another joint European society project.

The incidence of peripheral bone infection in the developed countries is less than 2% per year [6], but also higher incidence rates (2–4%) have been reported after surgical care of an open or closed fracture [7]. The incidence may even be up to 19% when trauma surgery takes place in an acute setting with possibly contaminated open fractures and concomitant soft tissue injuries [8, 9]. The incidence further increases in immunocompromised hosts, for example due to other diseases (HIV, autoimmune diseases), treatment (chemotherapy, immunosuppressive therapy), drug or alcohol abuse, or infectious root-canalled teeth [2].

In the acute phase after surgery, infection can usually easily be recognized by clinical examination (fever, redness, swelling, wound leakage, pain and disability of the affected body part). In the later phases, there can be clear signs of disease (fistula, purulent discharge), but often signs are subtler (slightly elevated temperature of the skin, diffuse pain) or not present at all and diagnosis may be very difficult at times. Peripheral bone infections are a serious healthcare threat due to several factors: the difficulty in making an early diagnosis (especially in low grade, chronic infections), treatment duration is long-lasting, often multiple surgical interventions are necessary, recurrence rate is high, and the impact on daily life for the affected patient is strong. Furthermore, when hematogenous spread occurs, PBI can even be life--threatening. Therefore, accurate diagnosis should be settled as early as possible, in order to promptly start an appropriate treatment and to avoid serious complications.

The diagnostic problem in PBI is that there is no single routine test available that can detect an infection with sufficiently high diagnostic accuracy. Mostly, a combination of clinical, laboratory, microbiological, and imaging tests is performed based on personal experience, available techniques, and expertise in the institute and financial aspects. Unfortunately, all available diagnostic tools have their limitations.

Current recommendations for diagnosing peripheral bone infection are scarce and all previous literature regarding the subject has certain limitations and shortcomings, such as solely based on expert opinions and/or local consensus meetings and not strictly focused on PBI. Moreover, they are affected by several shortcomings: absence of multidisciplinary approach, failure to provide a comprehensive diagnostic flowchart, and/or lack of inclusion of up-to-date diagnostic technology. International evidence-based guidelines in choosing the most accurate diagnostic strategy for PBI are lacking. Qualified members of the European Association of Nuclear Medicine (EANM), European Society of Radiology (ESR), European Society of Bone and Joint Society (EBJIS), and European Society of Clinical Microbiology and Infectious Diseases (ESCMID) had already developed a multidisciplinary approach to design a diagnostic flowchart for the management of PBIs [6].

An expert panel consisting of nuclear medicine physicians, infectious diseases specialists, radiologists, and orthopedic surgeons met on the occasion of the 20th congress of the EANM. The meeting led to the recognition amongst members of EANM, EBJIS, ESR, and ESCMID that a practical evidence-based guideline for diagnosing peripheral bone infections was needed. A homogeneous management of PBIs diagnosis trough a co-ordinated international, multi-disciplinary guidance would improve the sensitivity of diagnosis and consequently lead to a better outcome of patients with PBIs.

We therefore performed a systematic review of the articles published on this topic, in order to provide this consensus document on the diagnostic management of adult patients with PBIs, with special emphasis on radiologic and nuclear-medicine techniques. Our recommendations have been drawn up so as to be useful for a wide range of health care professionals, especially for radiologists, nuclear medicine physicians, infectious diseases specialists, and orthopedics.

## Methods

### Working group

After several joint symposia and the publication of the expert-based guidelines, we recognized that a multidisciplinary evidence-based guideline for diagnosing peripheral bone infections was needed. This joint society project started in 2015 and a working group was created with delegates from four European societies: the European Association of Nuclear Medicine (EANM), the European Bone and Joint Infection Society (EBJIS), the European Society of Microbiology and Infectious Diseases (ESCMID), and the European Society of Radiology (ESR). The delegates first met in Vienna (November 2015) to define the statements and after that in Rome (February 2016) to refine the statements and to define the final statements, based on available evidence that was circulated first among all participants. Finally, all delegates approved the final version of each statement.

### Statements, literature search, and scoring system

Uniform statements were addressed for each topic, with the aim of positioning all diagnostic procedures in a commonly agreed and evidence-based diagnostic flowchart. Literature search for the statements is described in Appendix 1. All included papers per statement were thoroughly read and analyzed and a “Level of Evidence” was provided in consensus with all delegates for each paper according to the documents for levels of evidence provided by OCEBM [10]. Each consensus statement is followed by a level of evidence, defined by the average of the levels of evidences of each included paper, and a short comment derived from analysis of the relevant literature. Statements are intended to be read in context with qualifying comments and not read in isolation.

## Available diagnostic methods for PBI

### Clinical assessment, symptoms, and signs of peripheral bone infection

In acute cases, individuals may report a generalized feeling of illness, loss of appetite, fatigue, nausea, and fever. Pain is the most common local symptom, and there may be a reduced ability to use the affected body part. A history of recent trauma, surgery, or infection of another organ (i.e., lungs, bladder) can be present. Individuals with chronic osteomyelitis may have a history of an acute episode. The patient may have an underlying immune system disease or peripheral vascular disease. The patient must be questioned about intravenous drug abuse and alcoholism. A complete medical history should be obtained, including all current and prior illnesses and injuries [2]. On physical examination, there can be clear signs of disease, like a direct fistula to the bone and purulent discharge, but often signs are more subtle like slight erythema and swelling or sub-febrile temperature and diffuse pain. Fever, signs of dehydration or other signs of blood infection (sepsis) may be evident. Range of motion of joints may be reduced [11]. In analogy with ulcers in patients with a diabetic foot pointing to osteomyelitis, ulcers in the tibia often reflect osteomyelitis. Therefore a probe-to-bone test can be helpful in establishing the diagnosis of PBI. In this test, the physician palpates the bone with a metal probe. This simple bedside procedure is based on the concept that if the probe can reach bone, so can infectious bacteria [Lavery 2007].

### Value of laboratory parameters

The specificity of elevated laboratory parameters of inflammation (white blood cell count, erythrocyte sedimentation rate (ESR), C-reactive protein (CRP), and procalcitonin) in differentiating PBI from other clinical conditions is low. In adult patients with PBI, ESR and CRP may be elevated, but on the contrary white blood cell counts is often in the normal range. Therefore, these parameters are not able to sufficiently discriminate the presence or absence of infection. However, a sharp increase of ESR and CRP may be helpful in confirming the diagnosis of PBI in the clinical context of a high level of suspicion [12] and may therefore influence the choice of imaging modality to be performed first. Also, serum procalcitonin may be used as a diagnostic marker for PBI [13]. Blood cultures should be performed for both aerobic and anaerobic germs in febrile patients in addition to biopsy, when possible.

### Bone biopsy

The gold standard for the correct identification of the causative microorganism of PBI is represented by culture of infected bone. Prior to collecting microbiological samples, any antibiotic regimen should be discontinued for 2 weeks, provided the progression of the disease allows this.

Biopsies should be taken under image guidance to provide representative samples. Bone is easily visualized with conventional X-ray and fluoroscopy. However, bone biopsies are generally conducted using CT guidance, which has the advantage of providing higher contrast resolution and better visualizaion of surrounding soft tissues, thus allowing for better evaluation of the exact location of the lesion and position of the needle. MRI guidance is rarely used for obtaining a bone biopsy. Because of the electromagnetic radiation, MRI-guided bone biopsy requires a special needle made of non-ferromagnetic stainless steel. Other disadvantages of MRI are longer procedural time and higher costs. MRI guidance should only be used in very selected cases like pediatric ones [14].

Bone biopsy samples should always be collected from a zone in which the bone structure is visibly inflamed. Tissue near visible bone or sequestra is informative. Collected pieces should be divided into two pieces for bacteriology and histology.

A minimum of three tissue samples should be collected. The more samples that are withdrawn, the less chance of an incorrect assessment due to contamination is reported. Whenever bone biopsies are done, the samples should be sent for aerobic and anaerobic cultures, cultures for mycobacteria and fungi should be performed in patients with clinical and epidemiological features supporting a suspicion for these etiologies. Samples collected directly from the skin should be avoided since these biopsies are often contaminated with skin microbes, leading to false-positive results. Histopathological analysis is essential for confirming or excluding the diagnosis of infection. Visualization of granulatomatous lesions with positive Ziehl–Neelsen staining may allow the diagnosis of mycobacterial infection (e.g., *Mycobacterium tuberculosis*).

Because bone biopsy is an invasive diagnostic method, several studies examined the diagnostic values of sinus tract cultures. However, these tract cultures are often contaminated with skin microbes, leading to a higher number of false-positive results. Superficial swab cultures showed inferior diagnostic values to sinus tract cultures and bone biopsy, and should not be used. New molecular methods can further improve the microbiological diagnosis [15].

### Radiological and nuclear medicine imaging methods and limitations

Several commonly used radiological and nuclear-medicine imaging methods are available (see Tables 1 and 2). An extensive description on the correct use of these techniques is provided in Appendix 2 [16–24]. The concerns on the use of ionizing radiation is described in Appendix 3 (https://ec.europa.eu/energy/sites/ener/files/documents/CELEX-32013L0059-EN-TXT.pdf, [25]).

**Table 1:** Pros and cons of advanced radiological techniques

	Ultrasound	CT	MRI
Pros	Useful for soft tissue extension and for biopsies Widely available Low cost No radiation burden	Useful in performing image-guided biopsies Widely available Medium cost	High diagnostic performance Feasible also with metallic implants in situ No need for contrast agent No radiation burden Widely available Medium cost
Cons	Cannot be used to diagnose bone infection	Possible artifacts due to metallic implants Lower diagnostic accuracy than MRI Radiation burden	Some false positivity due to edema

This table shows the pros and cons of radiological examinations to be performed as advanced diagnostic tests. From the data in literature, it emerges that MRI has higher accuracy than CT and should be preferred when available

**Table 2:** Pros and cons of advanced nuclear-medicine techniques

	Bone scan	Antigranulocyte scan	White blood cell scan	FDG-PET/CT
Pros	High sensitivity Useful as screening method in patients with low probability of an infection Widely available Low cost	High sensitivity and specificity (but lower than WBC scan) Widely available Medium cost SPECT/CT improves accuracy	High sensitivity and specificity, also with metallic implants in situ Not widely available Medium costs SPECT/CT improves accuracy	High sensitivity High specificity in patients without metallic implants and without recent surgery or fracture
Cons	Low specificity Moderate radiation burden	May induce Human anti-mouse antibody (HAMA) Moderate radiation exposure Late imaging time point necessary	Moderate radiation exposure Late imaging time point necessary Blood manipulation Needs approved laboratory and trained personnel	Difficult to differentiate between infection and inflammation with metallic implants are in situ, with recent surgery, or recent fracture Radiation exposure High cost

This table shows the pros and cons of nuclear-medicine examinations to be performed as advanced diagnostic tests

## Consensus statements

All performed PICOs for the statements and the papers finally included for the level of evidence are mentioned in Appendix 4.

1. **Patients presenting with clinical and radiological signs of peripheral bone infection or a positive probe-to-bone test may require further diagnostic procedures.**

- **Level of evidence: 5**

In case of clinical and radiological suspicion of peripheral bone infection, further diagnostic testing can be indicated to reveal severity and extent of the infection. Patients with acute peripheral bone infection can present with local pain, swelling, erythema, and warmth at the site of infection, and systemic symptoms such as fever and general illness. If a fistula is present, a probe to the bone test can be performed. In diabetic foot, this is indicative of bone infection, however, there is no literature supporting that statement in PBI. In general, in the acute phase with clear clinical signs, advanced imaging is often not necessary.

2. **Fistula direct to the bone and purulent discharge are evidence of bone infection.**

- **Level of evidence: 5**

There are no articles that provide evidence for this statement. It is based on common medical reasoning; bacteria that normally are present as part of skin flora superficially spread and colonize the exposed bone thereby causing local infection.

3. **CRP, ESR, and WBC counts should always be performed in patients suspected of having peripheral bone infection for diagnostic purposes.**

- **Level of evidence: 4**

In patients with PBI, raised ESR and CRP can be present, even if inconsistently, and can orientate versus a diagnosis of infection. White blood cell counts are more rarely increased. In patients with contiguous pedal osteomyelitis, the positive predictive value of ESR in diagnosing osteomyelitis in patients without diabetes was 78%, and in those with diabetes was 81%, with a negative predictive value 58 and 31%, respectively [26]. The concentration of CRP might be more reliable for follow-up of the response to treatment [2]. The role of serum procalcitonin was also evaluated in patients with acute PBI and a cut-off of 0.4 ng/ml was found to be a sensitive and specific marker in the diagnosis of acute osteomyelitis [13].

4. **Blood cultures should be considered in patients with fever suspected of having peripheral bone infection for diagnosing the involved bacteria**

- **Level of evidence: 4**

Blood cultures are positive mostly in hematogenous osteomyelitis. The evidence supporting the role of blood cultures in diagnosing PBIs other than vertebral osteomyelitis in adult patients is scanty. In our systematic review of the literature, we found only one study that analyzed the role of bacteremia in patients with bone infection. Adult patients with bone infection, regardless of the mechanism involved (i.e., trauma, bone surgery, joint replacement, bone damage resulting from vascular and/or cutaneous lesions) were included if blood cultures results were available. The authors found that bacteremia occurred in nearly 20% of the patients presenting with bone infection [27]. In this study, also patients with vertebral osteomyelitis were included and those were more frequently affected by bacteremia (53% patients had positive blood culture). Patients with bone infection associated with skin and soft tissue infections had positive blood cultures in 19% of cases; patients with osteosynthesis and open or closed fracture had positive blood cultures in 7% of cases. Importantly, only 41% of patients with bacteremia presented fever; this might suggest that blood cultures should be performed in patients with PBI irrespective of the presence of fever.

5. **Conventional radiography is the first imaging modality to be performed in patients suspected of having peripheral bone infection for diagnosis and follow-up.**

- **Level of evidence: 3**

Conventional radiography should always be performed first, as it may suggest correct diagnosis, exclude other pathologic conditions, and be correlated with other modalities. It is cheap and widely available. Bone changes are seen usually after 7–10 days from symptom onset, when 30–50% bone mass has been lost. Soft tissue swelling can be the only finding at first. Then, bone resorption and osteolysis can be seen. Last, periosteal reaction and bone formation can be detected. Sensitivity and specificity of conventional radiography in detection of acute osteomyelitis ranges between 43 and 75% and 75–83%, respectively [16, 17]. In anatomically complex locations (e.g., shoulder, pelvis) CT may replace conventional radiographs.

6. **In case of clinical and radiological signs of peripheral bone infection, bone biopsy is the reference standard for confirming infection and identifying the causative microorganism.**

- **Level of evidence: 4**

Patients presenting with clinical and radiological signs of peripheral bone infection may undergo bone biopsy to detect infection and identify the causative microorganism. Evidence is, however, conflicting and of low level.

Bernard et al. reported that surgical bone biopsy through the sinus tract had a sensitivity of 87%, a specificity of 79%, and a diagnostic accuracy of 82.5%, compared to those of surgical bone biopsy through a clinically uninfected area [28]. In a study analyzing 44 biopsy procedures performed in patients with osteomyelitis underlying an open wound, the large majority of biopsy cultures grew a bacterial isolate [29]. In a further prospective study including 100 patients with chronic osteomyelitis, bone cultures allowed etiologic diagnosis in 94% of cases; in the remaining cases, a diagnosis of OM was performed by histopathology analysis [30]. The role of culture of bone specimens obtained through a surgical biopsy has been confirmed also by other studies [31–33]. On the contrary, Wu et al. found a low rate of positivity for cultures obtained by imaging-guided biopsy in patients with OM [34].

Different modalities are available for obtaining a bone specimen, such as open bone biopsy, fine needle aspiration (FNA), and needle puncture. Bone biopsy is usually performed during the surgical debridement procedure. However, it can worsen peripheral vascular disease and neuropathy. Advantages of FNA over bone biopsy are that this procedure is less disruptive to bone and allows multiple samples to be taken [35]. In case of an ulcer, needle puncture is performed through normal skin surrounding the ulcer. This procedure is minimally invasive, easily performed, and has a greatly reduced risk of contamination [36]. With all of these modalities, care must be taken to prevent the infection from spreading to uninfected bone.

7. **In case of clinical and radiological signs of peripheral bone infection, sinus tract cultures and/or superficial swab cultures should be discouraged in the diagnostic work-up; bone biopsy is the gold standard.**

- **Level of evidence: 4**

Because bone biopsy is an invasive diagnostic tool, several studies aimed to examine the diagnostic value of sinus tract cultures and deep tissue cultures.

In our systematic review, we found five studies reporting that sinus tract cultures are inappropriate to identify the bone pathogen in osteomyelitis (https://ec.europa.eu/energy/sites/ener/files/documents/CELEX-32013L0059-EN-TXT.pdf, [2–32, 37]). In a study retrospectively analyzing 50 patients with chronic osteomyelitis, the concordance between bone and non-bone specimens was 28%. Moreover, cultures from non-bone specimens were false-negative in 52% of cases and false-positive in 36% [31]. In a further prospective study including patients with chronic osteomyelitis, the overall sensitivity (50.9%), specificity (20%), predictive value (47.5%), and concordance of sinus tract specimens with intraoperative bone specimens (41.4%) were very low [32]. Khatri et al. reported a poor correlation between wound and bone isolates [29]. In a prospective study, 100 adult patients with chronic osteomyelitis (excluding those with diabetic foot and decubitus ulcers) were included, at least one non-bone and one bone specimen were taken from each patient for microbiological analysis. Bone cultures grew a microorganism in 94% of cases. Importantly, the authors found that cultures of non-bone and bone specimens were microbiologically concordant in only 30% of patients. Slightly better concordance was found in chronic osteomyelitis caused by *Staphylococcus aureus* (42%) [30].

Only a single study reported different findings. In a large prospective non-randomized trial, in 140 patients with a cutaneous sinus tract, four microbiological samples were taken: two consecutive sinus tract cultures with bone contact (at different times), one surgical bone biopsy through the sinus tract, and one surgical bone biopsy through an uninfected area outside the sinus tract. The highest diagnostic accuracy rates in case of monomicrobial osteomyelitis with sinus tract were found with two concordant tract cultures with bone contact (94%). For polymicrobial infections, the accuracy was somewhat lower (79%). Even if this study supported reliability of two consecutive sinus tract cultures, the authors concluded that consecutive deep sinus tract specimens should not replace bone cultures in situations where a biopsy can readily be obtained. Bone culture remains the reference standard for the microbiological diagnosis of osteomyelitis; consecutive deep sinus tract cultures may be used when a biopsy cannot be performed [28].

8. **Antibiotic therapy should be discontinued before biopsy**

- **Level of evidence: 5**

In clinical practice, the dogma that antibiotics should be withheld before obtaining of microbiological cultures is well recognized and is based on the assumption that antibiotic exposure decreases the probability of bacterial identification from cultures. Obtaining an etiological diagnosis of PBI is extremely important for choosing the appropriate antimicrobial treatment. The antibiotic treatment should be guided by microbiological findings not only in order to improve the outcome of patients with PBI but also for limiting the use of unnecessary antibiotics and consequently limit the adverse effects and the ecological impact of antibiotics. As a consequence, all efforts should be made in order to obtain a microbiological diagnosis. No studies specifically assessing the impact of antibiotic exposure on the rate of positivity of bacterial cultures from bone biopsies in patients with PBIs have been published. In our systematic review, we found only three studies that reported some data on the impact of antibiotic exposure. Wu et al. found no significant differences in the culture positivity rate with regard to antibiotic therapy before image-guided biopsy [34]. Similarly, no significantly different diagnostic accuracy of microbiological cultures was found by Bernard et al. in patients with concomitant antibiotic treatment as compared with those without concomitant antibiotics [28]. In another study, no significant effect of prior antibiotic therapy on rate of positivity of bacterial cultures was found [29]. However, all of these studies were not designed with the purpose of investigating the issue of prior antimicrobial therapy, were cohort studies, and included a small number of patients.

Even if published studies were not able to demonstrate an effect of previous antibiotic therapy on the rate of microbiological culture positivity, considering the limitation of studies and the rationale for such recommendation, we recommend that discontinuing or postponing antibiotics when feasible is reasonable. Antimicrobial therapy should not be withheld in patients with impending sepsis or hemodynamic instability. The optimal duration of antibiotic-free time before bone biopsy has not been established.

9. **CT should be used as an adjunct to conventional radiographs in complex anatomic areas and is useful to detect bone sequestra.**

- **Level of evidence: 4**

CT has the highest image resolution in the evaluation of peripheral bone. Thus, it is indicated in the evaluation of complex anatomic area, such as the shoulder and the pelvis. It can be used to detect small foci of gas, and areas of cortical erosion and destruction. CT is the modality of choice in areas of complex anatomy for the detection of bone sequestra, mainly occurring at later stages of osteomyelitis [18].

10. **Non-contrast MRI has high diagnostic performance in detecting peripheral bone infection.**

- **Level of evidence: 2**

MRI has several advantages in the evaluation of peripheral bone infection. Not only it is able to evaluate infection of the bony component with high diagnostic performance, but it can also detect the presence and evaluate the extent of associated soft tissues abnormalities, such as muscular involvement or abscess formation. Non-contrast MRI has a sensitivity between 88 and 98%, a specificity between 70 and 96%, and a diagnostic accuracy of 81–86% in the diagnosis of peripheral osteomyelitis [19–21]. Apart from detecting bone infection, MRI can also optimally differentiate other conditions which clinically mimic infection including primary benign and malignant bone tumors, with the exception of cortical bone lesions (e.g., osteoid osteoma).

11. **Intravenous administration of gadolinium-based contrast agents does not increase the diagnostic performance of MRI in peripheral bone infection**

- **Level of evidence: 2**

Intravenous gadolinium-based contrast administration may help to better define the presence and extent of soft tissue abscesses in acute peripheral bone infection but it does not improve diagnostic performance [38]. It may also help avoid overstaging by better differentiating osteomyelitis from surrounding edema.

12. **The presence of a metallic implant/fixation device is not a contraindication to perform MRI in patients with suspected peripheral bone infection.**

- **Level of evidence: 5**

The presence of a metallic implant or metallic fixation device does not represent a contraindication to MRI. Traditionally, metallic implants were considered as potentially limiting the outcome of a MRI examination due to the high amount of susceptibility artifacts that could be generated by the metal itself. More recently, the advent of implants made with less ferromagnetic alloys and technical advancements of MR sequences (metal artifact reduction sequences [MARS], slice encoding for metal artifact correction [SEMAC], and multi-acquisition with variable-resonance image combination [MAVRIC]) made MRI fully feasible in patients with joint implants, with artifacts mostly limited to the area of the implant itself. However, some limitations to prosthesis geometry persist, affecting the assessment of the joint–prosthesis interface, and no papers are available on the topic; but this statement is made based on evidence published for joint prosthesis, a field with comparable issues.

13. **Three-phase bone scintigraphy is a sensitive technique in patients suspected for peripheral bone infection although not highly specific.**

- **Level of evidence: 2**

All included papers (meta-analyses and systematic reviews) for this statement report a similar high sensitivity, but low specificity [39–43]. The difference between sensitivity and specificity can be explained by the non-specific signs of peripheral bone infection on bone scintigraphy being increased vascularity during the first two phases and increased bone uptake at the late images. These three phases can also be positive because of other reasons, such as post-traumatic changes, fracture healing, recent surgery, etc. This explains the lower specificity especially in the post-traumatic or post-surgery setting. When using the three-phase bone scan, SPECT/CT is advised in the late phase for exact localization of the osteoblastic activity.

14. **White blood cell (WBC) scintigraphy and antigranulocyte antibody (AGA) scintigraphy have similar high diagnostic accuracy for diagnosis of peripheral bone infection.**

- **Level of evidence: 2**

Out of the 54 papers retrieved from the PICO for this statement, 14 were eventually included, only being clinical controlled studies and systematic reviews. These papers report a similar diagnostic accuracy for both WBC and AGA scintigraphy [42, 44–56]. However, the acquisition and interpretation criteria adopted are not always the same, making the comparison difficult between the used radiopharmaceuticals. The whole IgG (besilesomab) has a different biodistribution in vivo than the Fab fragment (sulesomab) and much more similar to radiolabeled white blood cells [57]. In addition, it is important to mention that two reports raise some questions about the specificity of binding of anti-granulocyte Fab fragment (sulesomab, Leukoscan®) to tissue infiltrating granulocytes [54, 58]. These two reports do not, however, bring sufficient scientific evidence for this assumption and should therefore be considered as “expert opinions” and not entirely pertinent to this statement. Therefore, considering only papers dealing with diagnostic accuracy in peripheral bone infections, the level of evidence for this statement is 2.

15. **Pre-test probability of infection should be considered for choosing between three-phase bone scan and WBC scintigraphy (fractures, recent surgery, osteosynthesis, highly positive serological tests).**

- **Level of evidence: 5**

Despite an extensive literature search, no original papers or review articles could be retrieved that describe the importance of the pre-test probability for a peripheral bone infection to choose between three-phase bone scintigraphy or white blood cell scintigraphy. However, based on daily clinical practice, one always considers the pre-test probability before considering a particular imaging modality. Because three-phase bone scan has a high sensitivity, but low specificity especially in ‘violated’ bones, we recommend performing WBC scintigraphy in patients with suspicion of infection after recent surgery or in the setting of metallic hardware in situ, or recent fractures. In contrast, in patients with low likelihood for infection based on clinical and biochemical parameters and non-violated bone, a three-phase bone scan can be recommended to exclude an infection because of high sensitivity, low cost, and wide availability. The level of evidence for this statement is 5, and should be regarded as an expert opinion.

16. **a.** ^**18**^ **F-FDG-PET has high diagnostic accuracy in peripheral bone infection without fracture and osteosynthesis.**
**Level of evidence: 2**

- **b. WBC scintigraphy is the preferred nuclear-medicine imaging technique of choice in patients suspected of peripheral bone infection with recent fracture of hardware in situ.**
- **Level of evidence: 2**

All included papers (both original and reviews) agree that FDG-PET is a promising and accurate imaging technique for peripheral bone infection without hardware in situ [6, 39, 51, 53, 59–64] but they also agree that there is not sufficient evidence for using this modality as the current reference standard. Most studies are performed in patients with chronic osteomyelitis, so the role of FDG-PET in the acute phase is still unknown. This number of cases is still too small to conclude that FDG-PET is superior to WBC scintigraphy in PBI [63]. At the moment, both techniques (FDG-PET and WBC scintigraphy) can be used with the same diagnostic accuracy in patients with peripheral bone infection without recent fracture and/or without metallic hardware in situ.

In patients with hardware in situ or with a recent fracture, the number of patients imaged with FDG-PET is even more limited. Only three studies exist that included > 10 patients with metallic hardware in situ [64–66]. The retrospective study of Wenter et al. included the largest number of patients with hardware in situ [60], leading to a diagnostic accuracy of 86%. Much more data is available for WBC scintigraphy in patients with recent fractures or metallic hardware in situ and this technique is still the preferred nuclear-imaging technique in patients suspected of peripheral bone infection with recent fracture or hardware in situ.

17. **Hybrid SPECT-CT WBC imaging can be performed for exact localization of infection site.**

- **Level of evidence: 2**

Since the development of hybrid imaging techniques such as SPECT-CT, in general diagnostic accuracy has significantly been increased due to the synergistic effect of combining pathophysiology with anatomy. However, in the recommendations about acquisition and interpretation of WBC scintigraphy, only planar images on several time points are considered. Theoretically, adding the SPECT-CT in positive cases would also for this indication lead to a higher diagnostic accuracy.

Even though limited data is available (only eight included papers, of which four were review articles), the advantage of SPECT/CT over planar images is clear in all papers [23, 67–73]. This is well known for other indications; the better resolution of SPECT and the added CT component for anatomy leads to higher diagnostic accuracy. All included papers agree in their opinion: in patients with suspected peripheral bone infection, SPECT/CT should be performed when there is positivity on the planar WBC images. Adding SPECT/CT leads to better differentiation between bone involvement of the infection and soft tissue infection and leads to better characterization of the extent of the infection. The CT component itself can help in anatomical difficult cases, e.g., after a major trauma.

18. **When having a suspicion for hematogenous spread of the infection,** ^**18**^ **F-FDG-PET/CT is the first imaging modality of choice.**

- **Level of evidence: 5**

Although an extensive PICO was performed with trying many search terms, hardly any articles were found that describe the role of FDG-PET when having suspicion of dissemination in patients with a peripheral bone infection. Only an overview paper about pitfalls in infection imaging was retrieved (expert opinion) [74]. However, from daily clinical practice, the benefits of an easy whole-body imaging technique to visualize all infectious lesions within a patient are clear. The technique is highly sensitive, although not very specific, easy to perform, and comfortable for the patient. So, although evidence is low, we recommend the use of FDG-PET/CT when having suspicion of hematogenous spread of infection.

## Evidence-based diagnostic flowchart

**Fig. 1:**
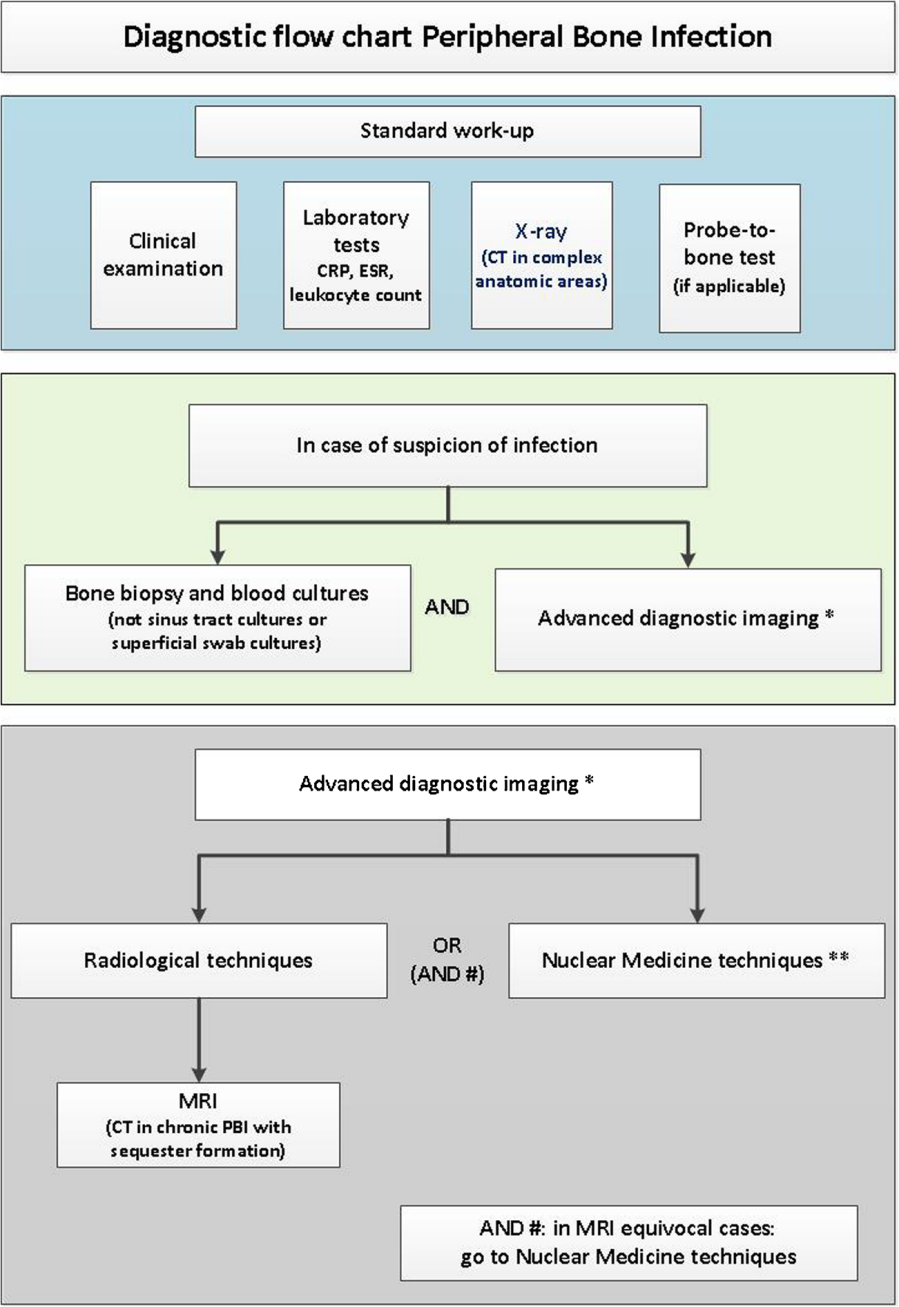
The suggested initial diagnostic steps to undertake in the suspicion of PBI, based on published evidence. Of course not all steps are required, this may change in the individual patient. Some steps can of course be repeated when necessary. Serological tests can be performed over time since the trend to increase or decrease is more important than a single value. At this moment, there is not enough clinical evidence to support the use of one advanced diagnostic imaging technique above the other. There is a lack of studies with large patient numbers and there are hardly no comparative studies. Therefore, the choice of which advanced diagnostic modality to be used first depends on several factors, such as experience of the imaging specialist, costs, availability, radiation burden and local expertise (see also Tables 1 and 2). In many hospitals, MRI is considered as the first advanced imaging modality in daily practice, mainly because there is no radiation involved. In patients with metallic hardware, however, there is sufficient literature to support a preferential use of white blood cell scintigraphy

**Fig. 2:**
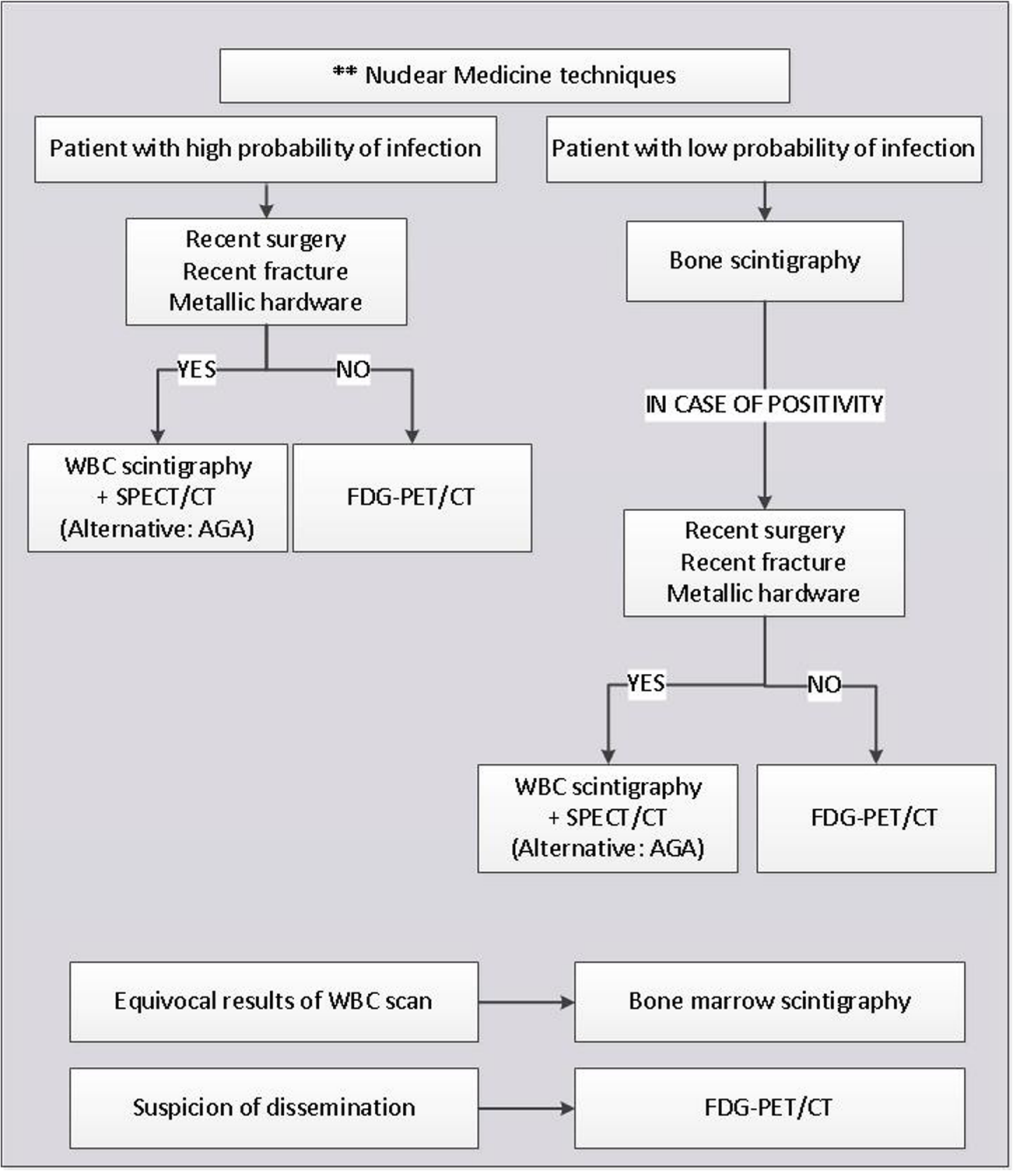
The suggested path to undertake when nuclear-medicine techniques are considered in the suspicion of PBI, based on published evidence and expert opinion. This flowchart indicates when to use which nuclear imaging modality and is based on scientific evidence as mentioned in the statements

Based on the above-mentioned statements and evidence from published literature, we have developed the diagnostic flowchart shown in Figs. 1 and 2.

In some cases, the flow was integrated by consensus opinion amongst the experts, since not all steps are always clearly deducible from literature or from level 1–2 articles. The flowchart does not take into consideration socio-economical factors and the availability of diagnostic methods. It also presumes that all exams are performed at their best (possibly following procedural guidelines published by each society, when available) and by expert professionals.

The flowchart starts with the suggested initial steps to undertake when having a suspicion for PBI, based on the aforementioned evidence. Clinical examination, laboratory tests, and conventional X-ray should be performed in all cases. Laboratory tests should be performed over time since the trend to increase or decrease is more important than a single value. The probe-to-bone test can be helpful in some cases to establish the diagnosis PBI, e.g., when ulcers in the tibia are present.

When still having a suspicion at this stage, image-guided bone biopsy should be performed to detect infection and identify the causative micro-organism. Sinus tract cultures can cause false-positive results due to contamination, and superficial swab cultures have a lower diagnostic than bone biopsy. Besides the bone biopsy, advanced diagnostic imaging tests should be performed. Since there are not enough well-designed studies (with a level of evidence 1) available that directly compare radiological with nuclear-medicine techniques, we decided to split the imaging techniques into radiological and nuclear-medicine techniques. When choosing an advanced diagnostic imaging test, a further stratification should be performed based on the pre-test probability of infection. Based on local experience, one can decide to start with radiological techniques (MRI has the best diagnostic accuracy) and use nuclear-medicine techniques in case of equivocal MRI results. If a center is very experienced in nuclear-medicine modalities, one can decide to start with these techniques. Which nuclear-medicine modality should be used first depends on the following: is there a low or a high probability of infection? Was there recent surgery? Was there a recent fracture? Is there metallic hardware in situ? For the answers to these questions, see the nuclear part of the flowchart and tables.

## Conclusions and final recommendations

This is the first proposal of a diagnostic flowchart in patients with suspicion of a peripheral bone infection based on evidence available from literature. However, since no large multicenter prospective comparative studies exist, we should conclude that the available evidence is mostly limited. Therefore, we also had to include expert opinion (by consensus) in the presented flowchart. We think that the presented flowchart can be a first step in many centers and can form a basis to start large prospective studies to directly compare the different advanced imaging techniques to develop future flowcharts.

## Electronic supplementary material

ESM 1 (DOCX 15 kb)

ESM 2 (DOCX 20 kb)

ESM 3 (DOCX 15 kb)

ESM 4 (DOCX 31 kb)

## Abbreviations

PBI: Peripheral bone infection
CRP: C-reactive protein
ESR: Erythrocyte sedimentation rate
WBC: White blood cells
PICO: Population/problem – Intervention/indicator – Comparator – Outcome
CT: Computerized tomography
EANM: European Association of Nuclear Medicine
ESR: European Society of Radiology
EBJIS: European Bone and Joint Infection Society
ESCMID: European Society of Clinical Microbiology and Infectious Diseases
OCEBM: Oxford Centre for Evidence-based Medicine
MRI: Magnetic resonance imaging
FDG: ^18^F-fluorodeoxyglucose
^99m^Tc: Technetium
PET: Positron emission tomography
AGA: Anti-granulocyte antibodies
mAbs: Monoclonal antibodies
SPECT: Single-photon emission computerized tomography
US: Ultrasound
HMPAO: Hexamethylpropylene-amine oxime
T/B: Target-to-background ratio
Oxine: 8-hydroxyquinoline
MARS: Metal artifact reduction sequences
MAVRIC: Multiacquisition with variable-resonance image combination
SEMAC: Slice encoding for metal artifact correction
