# Supplementary material for: Consensus document for the diagnosis of peripheral bone infection in adults: a joint paper by the EANM, EBJIS, and ESR (with ESCMID endorsement)
Source: Eur J Nucl Med Mol Imaging. 2019 Jan 24;46(4):957–70. doi: 10.1007/s00259-019-4262-x (PMC6450853; doi:10.1007/s00259-019-4262-x)
Supplement: Supplementary file 1 — (DOCX 15 kb) [file 259_2019_4262_MOESM1_ESM.docx]

**Appendix 1. Literature search.**

***Literature search***

Panel members thoroughly reviewed the literature pertinent to each of the questions and thereby evaluated the diagnostic accuracy of each diagnostic technique. Search terms were defined in agreement with all delegates from the four participating societies. Articles presenting data pertaining to the diagnosis of PBIs were identified through computerized literature searches using PubMed/MEDLINE (National Library of Medicine Bethesda MD) and Scopus databases and by reviewing the references of retrieved articles. For the development of the background section we also reviewed articles describing the epidemiology of PBIs. Index search terms were selected according to the Oxford Centre for Evidence based Medicine, as reported in appendix 1. The search was restricted to full articles published in English and including adult patients (>16 years of age). For papers dealing with imaging techniques the time-period from January 2000 on was chosen since important developments in existing camera systems and imaging techniques were achieved after the year 2000. No attempt was made to obtain information on unpublished studies. Since data from randomised clinical trials were expected to be limited, we also reviewed non-randomised controlled clinical trials, cohort and case-control studies. Single case reports and reviews, were excluded as well as studies including less than 10 patients. Meta-analyses were included if performed according to Cochrane criteria and when they included eligible papers. Inclusion of the papers per statement was based on a PICO (Population/problem – Intervention/indicator – Comparator – Outcome) question to search for evidence after converting the PICO question into a search strategy. This strategy is described extensively by the Oxford Centre for Evidence-based medicine (OCEBM) [11].
