# Supplementary material for: Consensus document for the diagnosis of peripheral bone infection in adults: a joint paper by the EANM, EBJIS, and ESR (with ESCMID endorsement)
Source: Eur J Nucl Med Mol Imaging. 2019 Jan 24;46(4):957–70. doi: 10.1007/s00259-019-4262-x (PMC6450853; doi:10.1007/s00259-019-4262-x)
Supplement: Supplementary file 2 — (DOCX 20 kb) [file 259_2019_4262_MOESM2_ESM.docx]

**Appendix 2 Imaging techniques.**

***Radiologic imaging methods and limitations***

Radiological imaging is usually the first modality used to assess patients with peripheral bone infection. In particular, conventional radiographs have been traditionally used as the first diagnostic approach. However, conventional radiographs generally only become positive when at least 30% to 50% bone mass has been lost, making early diagnosis unlikely. However, they are still used to have a panoramic evaluation of the bone structure overall and to detect the presence of associated findings [17, 18]. Computed tomography (CT) may play a role in anatomically-complex zones, such as the shoulder or the pelvis. Its use is usually reserved in patients with chronic osteomyelitis to detect the presence of bone sequestra [19] but is not particularly used in an acute context. Magnetic resonance imaging (MRI) has high diagnostic performance in diagnosing PBI (88-98% sensitivity, 70-96% specificity, and 81-86% accuracy) [20-22]. MRI has three main advantages. First, it does not use ionizing radiation, making it safe for patients from this perspective. Then, most cases can be ruled out without intravenous administration of contrast agents, which can be used in selected cases to better define the involvement of surrounding soft tissues. These two important features are extremely important in these kind of patients, who generally need repeated examinations over time. Last, technologic evolution allowed developing new specific imaging sequences (Metal Artefact Reduction Sequence, MARS) which allow reducing considerably artifacts related to the presence of metallic implants for certain prosthesis geometries, which thus no longer are a factor that may limit the outcome of a MRI examination should the sequences be available on the particular scanner (not every scanner is equipped with the MARS sequence). Also for CT, metal artifact reduction reconstruction techniques are advancing.

***Nuclear medicine imaging methods and limitations***

*Bone scintigraphy*

Bone scintigraphy is performed by intravenous administration of diphosphonates labelled with ^99m^Tc. Generally, the bone scan consists of three phases (perfusion, blood pool, and late phase with incorporation of the radiopharmaceutical into the bone matrix). Bone scintigraphy can be used as a first screening method to exclude the presence of peripheral bone infection in case of negativity, because of its good availability, relatively low costs and high negative predictive value. After recent fracture and/or surgery and in the presence of a high suspicion, the role of bone scintigraphy is negligible, since the specificity is rather low and uptake can be seen in all sites of increased bone metabolism irrespective of the underlying disease. Recently, the EANM Bone and Joint Committee published a procedural guideline on how to perform bone scintigraphy [23]. Nowadays also the PET tracer ^18^F-sodium fluoride may be used, but its widespread use is still limited due to limited availability, high costs, reimbursement issues, and inabilities to acquire perfusion and blood pool images in a practical setting.

*White blood cell scintigraphy*

According to different biodistribution and kinetics of white blood cells (WBC) in blood, bone marrow, inflammation and infection, preferably 3 sets of images should be acquired: “early” images (between 30 minutes and 1 hour after injection), “delayed” images (2-4 h after injection) and “late” images (20-24 h after injection). A scan should be considered positive for an infection when there is an increase in size or intensity with time. When using the correct acquisition protocols and interpretation criteria this technique leads to excellent overall diagnostic accuracy and this imaging modality is considered the reference standard nuclear diagnostic technique for peripheral bone infection (24). However, the complete study requires several scans over 2 days, blood manipulation is necessary and the technique is not available in each nuclear medicine center.

*Anti-granulocyte antibody scintigraphy*

As an alternative for WBC scintigraphy labelled anti-granulocyte antibodies (AGA) can be used. There are two antibodies commercially available: a Fab fragment, Sulesomab (Leukoscan®) and a whole murine IgG Besilesomab (Scintimun®). Image protocols differ between complete and fragmented antibodies. Images with complete antibody (Besilesomab) should be performed 2-4 h and 16-24 h post injection. Planar images and, in case of positivity followed by SPECT/CT, can be performed with the same acquisition protocol as WBC. Images with the fragmented antibody (Sulesomab) should be performed 1-3 and 6-8 h post injection.

*Bone marrow scintigraphy*

Bone marrow (BM) scintigraphy can be used in patients with suspected peripheral bone infection when WBC (or AGA) scans are doubtful. ^99m^Tc-colloids (colloids larger than 500 nm are recommended) are injected intravenously and images of the region of interest are acquired after a minimum of 30 minutes and a maximum time of 6 h post-injection. When there is concordance between WBC and BM scan (uptake in the same region) this is probably due to physiological bone marrow activity; discordant findings (positive on WBC scan, negative on BM scan) point to an infection.

*^18^F-fluorodeoxyglucose (FDG) PET*

The use of the glucose analogue FDG in infectious diseases increased significantly during the last years. This technique has many advantages: no blood manipulation, high spatial resolution, one imaging time point already 1 h after infection, etc. The main limitation however is that FDG is taken up both in inflammatory and infectious lesions and discrimination between both is difficult, especially when there is metallic hardware in situ or if there was a recent fracture and/or surgery. FDG-PET/CT as an alternative to WBC scintigraphy has been described by many authors as highly sensitive and specific for the diagnosis peripheral bone infection. However, there are still concerns whether the presence of fractures or osteosynthesis can induce false positive uptake of FDG that might reduce the diagnostic accuracy of the method. Currently, there are no clear interpretation criteria for declaring a FDG-PET as positive or negative for peripheral bone infection, and mostly diagnosis is based on subjective criteria and experience. In the chronic peripheral non-postoperative setting results are equal to WBC scintigraphy (25) with a potential advantage of FDG-PET related to the logistically easier imaging method, wide availability and high negative predictive value. In the acute setting, or with metallic hardware in situ, WBC scintigraphy is still preferred.

***Hybrid imaging techniques***

The advent of hybrid imaging technologies combining molecular/functional and anatomical information has significantly increased the diagnostic accuracy of conventional nuclear exams by increasing sensitivity and specificity and reducing the number of equivocal lesions. This hybrid technology has redefined the work-up of our patients and has influenced patient management. The hybrid imaging techniques has improved image properties because of (1) advances in detector designs and collimator modeling, inherent to newer devices, (2) incorporation of CT data into SPECT reconstruction, and (3) fusion of anatomic and functional data, allowingmore accurate localization and assessment of disease extent.

SPECT/CT should be an integral part of a conventional WBC scintigraphy mainly aiming to better distinguish bone from soft tissue infections and to more accurately assess the extent of the infectious process. PET/CT to combine (patho)physiology with anatomy is already considered the gold standard, the use of a PET only camera nowadays is considered obsolete.

More recently, the introduction of PET/MRI has emerged as a powerful diagnostic tool, but so far no reports have been published on its value in peripheral bone infection. The general advantages of PET/MRI compared to PET/CT will be a better evaluation of soft tissue due to the MRI part and the reduced radiation burden.

Finally, one should of course always keep in mind that the final decision for a particular imaging technique highly depends on local availability, time, costs and expertise.
