# Supplementary material for: Consensus document for the diagnosis of peripheral bone infection in adults: a joint paper by the EANM, EBJIS, and ESR (with ESCMID endorsement)
Source: Eur J Nucl Med Mol Imaging. 2019 Jan 24;46(4):957–70. doi: 10.1007/s00259-019-4262-x (PMC6450853; doi:10.1007/s00259-019-4262-x)
Supplement: Supplementary file 3 — (DOCX 15 kb) [file 259_2019_4262_MOESM3_ESM.docx]

**Appendix 3.**

***Concerns on the use of ionizing radiations***

Ionizing radiations represent a potential risk in patients undergoing radiological and nuclear medicine examinations in clinical practice. In 2014, the European Society of Radiology launched the EuroSafe Imaging Campaign to promote and strengthen medical radiation protection across Europe [http://www.eurosafeimaging.org/]. Similar concern has always been taken into consideration by the EANM with a significant reduction of administered activities to patients (also due to advances in technology), with regularly updated recommendations over the years.

On the basis of previous directives, the European Union issued a new document on basic safety standards for protection against the dangers arising from medical exposure to ionising radiation [26]. In Chapter VII, article 55, comma 1, the principle of justification of medical exposure is clearly stated. Here, the need of taking into account efficacy, benefits, and risks of alternative techniques not involving the use of ionizing radiation is clearly stated. This means that if an imaging modality without ionizing radiations has similar diagnostic performance of a modality using ionizing radiation, the first should be invariably preferred.

The principle of justification to medical exposure is clearly relevant and the referring clinician and the physician must always consider efficacy, benefits and risks as well as the possibility to obtain similar results by using alternative techniques not involving the use of ionizing radiation [27].
