# Supplementary material for: Consensus document for the diagnosis of peripheral bone infection in adults: a joint paper by the EANM, EBJIS, and ESR (with ESCMID endorsement)
Source: Eur J Nucl Med Mol Imaging. 2019 Jan 24;46(4):957–70. doi: 10.1007/s00259-019-4262-x (PMC6450853; doi:10.1007/s00259-019-4262-x)
Supplement: Supplementary file 4 — (DOCX 31 kb) [file 259_2019_4262_MOESM4_ESM.docx]

**APPENDIX 4. PICOs belonging to statements.**

1. **Patients presenting with clinical and radiological signs of peripheral bone infection or a positive probe-to-bone test require further diagnostic procedures.**

**Level of evidence: 5**

**P: bone infection OR osteomyelitis OR osteitis**

**I:** clinical signs OR radiological signs OR probe to bone test

**C: -**

**O: diagnostic accuracy OR sensitivity OR specificity**

**Search terms** (((((bone infection[Title/Abstract]) OR osteomyelitis[Title/Abstract]) OR osteitis[Title/Abstract])) AND ((Clinical signs OR radiological signs OR probe to bone test))) AND ((diagnostic accuracy OR sensitivity OR specificity))

**All three together: 620 articles**

**Included papers for thorough reading: 28 articles**

**Included papers after thorough reading: 0 articles**

1. **Fistula direct to the bone and purulent discharge are evidence of bone infection.**

**Level of evidence: 5**

**P: bone infection OR osteomyelitis OR osteitis**

**I: sinus tract OR fistula OR swab sample**

**C: -**

**O: diagnostic accuracy OR sensitivity OR specificity**

**Search terms (((sinus tract OR fistula OR swab sample))) AND ((bone infection[Title/Abstract] OR osteomyelitis[Title/Abstract] OR osteitis[Title/Abstract]))**

**All three together: 503 articles**

**Included papers for thorough reading: 5 articles**

**Included papers after thorough reading: 0 articles**

1. **CRP, ESR and WBC counts should always be performed in patients suspected to have peripheral bone infection for diagnostic purposes.**

**Level of evidence: 4**

P osteomyelitis OR bone infection

I c-reactive protein OR erythrocyte sedimentation rate OR white blood cells

C-

O diagnosis

**Search terms** (("osteomyelitis"[MeSH Terms] OR "bone infection"[All Fields]) AND ("diagnosis"[Subheading] OR "diagnosis"[All Fields] OR "diagnosis"[MeSH Terms]) AND (("c-reactive protein"[MeSH Terms] OR ("c-reactive"[All Fields] AND "protein"[All Fields]) OR "c-reactive protein"[All Fields] OR "c reactive protein"[All Fields]) OR "erythrocyte sedimentation rate "[All Fields] OR "white blood cells"[All Fields])) AND (("2000/01/01"[PDAT] : "2015/12/31"[PDAT]) AND "humans"[MeSH Terms] AND English[lang] AND "adult"[MeSH Terms])

**All three together:** 98

**Included papers for thorough reading:** 17

**Included papers after thorough reading:** 8

1. **Blood cultures should be considered in patients with fever suspected to have peripheral bone infection for diagnosing the involved bacteria**

**Level of evidence: 4**

P osteomyelitis OR bone infection

I blood culture

C-

O diagnosis

Search terms: ("osteomyelitis"[MeSH Terms] OR "bone infection"[All Fields]) AND ("diagnosis"[Subheading] OR "diagnosis"[All Fields] OR "diagnosis"[MeSH Terms]) AND (("blood"[Subheading] OR "blood"[All Fields] OR "blood"[MeSH Terms]) AND ("culture"[MeSH Terms] OR "culture"[All Fields] OR "cultures"[All Fields])) AND (("2000/01/01"[PDAT] : "2015/12/31"[PDAT]) AND "humans"[MeSH Terms] AND English[lang] AND "adult"[MeSH Terms])

**All three together:** 100

**Included papers for thorough reading:** 11

**Included papers after thorough reading:** 1

1. **Conventional radiography is the first imaging modality to be performed in patients suspected to have peripheral bone infection for diagnosis and follow-up.**

**Level of evidence: 3**

P: acute osteomyelitis OR acute bone infection AND adults NOT diabetes OR diabetic foot OR spine OR spondylodiscitis

I: xray OR plain film OR conventional radiography

C: -

O: diagnostic performance OR diagnosis OR accuracy

Search terms: ((((acute osteomyelitis OR acute bone infection AND adults)) NOT (diabetes OR diabetic foot OR spine OR spondylodiscitis)) AND (xray OR plain film OR conventional radiography)) AND (diagnostic performance OR diagnosis OR accuracy)

**All three together:** 37

**Included papers for thorough reading:** 19

**Included papers after thorough reading:** 2

1. **In case of clinical signs of peripheral bone infection, bone biopsy is the gold standard for confirming infection and identifying the causative microorganism.**

**Level of evidence: 4**

P: bone infection OR osteomyelitis

I: bone biopsy

C: -

O: diagnosis

Search terms: (("osteomyelitis"[MeSH Terms] OR "bone infection"[All Fields]) AND ("diagnosis"[Subheading] OR "diagnosis"[All Fields] OR "diagnosis"[MeSH Terms]) AND (("bone and bones"[MeSH Terms] OR ("bone"[All Fields] AND "bones"[All Fields]) OR "bone and bones"[All Fields] OR "bone"[All Fields]) AND ("pathology"[Subheading] OR "pathology"[All Fields] OR "biopsy"[All Fields] OR "biopsy"[MeSH Terms])))

All three together: 777 articles

Included papers for thorough reading: 42 articles

Included papers after thorough reading: 6 articles

1. **In case of clinical signs of peripheral bone infection, sinus tract cultures and/or superficial swab cultures should be discouraged in the diagnostic work-up; bone biopsy remains the gold standard.**

**Level of evidence: 4**

**P: bone infection OR osteomyelitis OR**

**I: sinus tract OR fistula OR swab sample**

**C: -**

**O: diagnosis**

Search terms: (("osteomyelitis"[MeSH Terms] OR "bone infection"[All Fields]) AND ("diagnosis"[Subheading] OR "diagnosis"[All Fields] OR "diagnosis"[MeSH Terms]) AND (("bone and bones"[MeSH Terms] OR ("bone"[All Fields] AND "bones"[All Fields]) OR "bone and bones"[All Fields] OR "bone"[All Fields]) AND ("pathology"[Subheading] OR "pathology"[All Fields] OR "biopsy"[All Fields] OR "biopsy"[MeSH Terms]))) AND (("2000/01/01"[PDAT] : "2015/12/31"[PDAT]) AND "humans"[MeSH Terms] AND English[lang] AND "adult"[MeSH Terms])

All three together: 777 articles

Included papers for thorough reading: 40 articles

Included papers after thorough reading: 6 articles

1. **Antibiotic therapy should be discontinued before biopsy**

**Level of evidence: 5**

**P: bone infection OR osteomyelitis**

**I: biopsy**

**C: -**

**O: diagnosis**

Search terms: (("osteomyelitis"[MeSH Terms] OR "bone infection"[All Fields]) AND ("diagnosis"[Subheading] OR "diagnosis"[All Fields] OR "diagnosis"[MeSH Terms]) AND (("bone and bones"[MeSH Terms] OR ("bone"[All Fields] AND "bones"[All Fields]) OR "bone and bones"[All Fields] OR "bone"[All Fields]) AND ("pathology"[Subheading] OR "pathology"[All Fields] OR "biopsy"[All Fields] OR "biopsy"[MeSH Terms]))) AND (("2000/01/01"[PDAT] : "2015/12/31"[PDAT]) AND "humans"[MeSH Terms] AND English[lang] AND "adult"[MeSH Terms])

All three together: 777 articles

Included papers for thorough reading: 40 articles

Included papers after thorough reading: 3 articles

1. **CT should be used as an adjunct to conventional radiographs in complex anatomic areas and is useful to detect bone sequestra.**

**Level of evidence: 4**

P: acute osteomyelitis OR acute bone infection AND adults NOT diabetes OR diabetic foot OR spine OR spondylodiscitis

I: computed tomography OR computed assisted tomography

C: -

O: diagnostic performance OR diagnosis OR accuracy

Search terms: ((((acute osteomyelitis OR acute bone infection AND adults)) NOT (diabetes OR diabetic foot OR spine OR spondylodiscitis)) AND (computed tomography OR computed assisted tomography)) AND (diagnostic performance OR diagnosis OR accuracy)

All three together: 203 articles

Included papers for thorough reading: 37 articles

Included papers after thorough reading: 3 articles

1. **Non-contrast MRI has high diagnostic performance in detecting peripheral bone infection.**

**Level of evidence: 2**

P: acute osteomyelitis OR acute bone infection AND adults NOT diabetes OR diabetic foot OR spine OR spondylodiscitis

I: magnetic resonance imaging

O: diagnostic performance OR diagnosis OR accuracy

Search terms: ((((acute osteomyelitis OR acute bone infection AND adults)) NOT (diabetes OR diabetic foot OR spine OR spondylodiscitis)) AND magnetic resonance imaging) AND (diagnostic performance OR diagnosis OR accuracy)

All three together: 139 articles

Included papers for thorough reading: 41 articles

Included papers after thorough reading: 3 articles

1. **Intravenous administration of Gadolinium-based contrast agents does not increase the diagnostic performance of MRI in peripheral bone infection.**

**Level of evidence: 2**

P: acute osteomyelitis OR acute bone infection AND adults NOT diabetes OR diabetic foot OR spine OR spondylodiscitis

I: magnetic resonance imaging AND contrast-enhanced OR contrast OR Gadolinium

C:-

O: diagnostic performance OR diagnosis OR accuracy

((((acute osteomyelitis OR acute bone infection AND adults)) AND (diabetes OR diabetic foot OR spine OR spondylodiscitis)) AND (magnetic resonance imaging AND contrast-enhanced OR contrast OR Gadolinium)) AND (diagnostic performance OR diagnosis OR accuracy)

All three together: 17 articles

Included papers for thorough reading: 7 articles

Included papers after thorough reading: 1 articles

1. **The presence of a metallic implant/fixation device is not a contraindication to perform MRI in patients with suspected peripheral bone infection.**

**Level of evidence: 5**

P: acute osteomyelitis OR acute bone infection AND adults NOT diabetes OR diabetic foot OR spine OR spondylodiscitis

I: magnetic resonance imaging AND metallic implant OR metal fixation device

C: -

O: feasibility OR diagnostic performance

Search terms: ((((acute osteomyelitis OR acute bone infection AND adults)) AND (diabetes OR diabetic foot OR spine OR spondylodiscitis)) AND (magnetic resonance imaging AND metallic implant OR metal fixation device)) AND (feasibility OR diagnostic performance)

All three together: 0 articles

1. **Three-phase bone scintigraphy is a sensitive technique in patients suspected for peripheral bone infection although not highly specific.**

**Level of evidence: 2**

P = osteomyelitis or osteitis

I = MDP or HDP or bone scintigraphy

C = -

O = diagnosis of infection

Search terms: (peripheral or bone or osteomyelitis or infection or osteitis) AND (MDP or HDP or bone scintigraphy) AND (diagnosis or bone or infection OR diagnostic or accuracy OR osteomyelitis)

All three together: 35 articles

Included papers for thorough reading: 15 articles

Included papers after thorough reading: 5 articles

1. **White blood cell (WBC) scintigraphy and antigranulocyte antibody (AGA) scintigraphy have similar high diagnostic accuracy for diagnosis of peripheral bone infection.**

**Level of evidence: 2**

1. **White blood cell (WBC) scintigraphy**

P = peripheral or bone or osteomyelitis or infection

I = wbc or scintigraphy or scan or radiolabel* white blood cells or radiolabel* leucocytes

C = -

O = diagnosis or bone or infection OR diagnostic or accuracy OR osteomyelitis

Search terms: (peripheral or bone or osteomyelitis or infection) AND (wbc or scintigraphy or scan or radiolabel* white blood cells or radiolabel* leucocytes) AND (diagnosis or bone or infection OR diagnostic or accuracy OR osteomyelitis)

All three together: 39 articles

Included papers for thorough reading: 15 articles

Included papers after thorough reading: 12 articles

1. **antigranulocyte antibody (AGA) scintigraphy**

P = peripheral or bone or osteomyelitis or infection

I = leukoscan or sulesomab or scintimun or besilesomab on MN3 or BW250/183 or BW250 or BW-250 or NCA95 or NCA90

C = -

O = diagnosis or bone or infection OR diagnostic or accuracy OR osteomyelitis

Search terms: (peripheral or bone or osteomyelitis or infection) AND (leukoscan or sulesomab or scintimun or besilesomab on MN3 or BW250/183 or BW250 or BW-250 or NCA95 or NCA90) AND (diagnosis or bone or infection OR diagnostic or accuracy OR osteomyelitis)

All three together: 14 articles

Included papers for thorough reading: 8 articles

Included papers after thorough reading: 5 articles

1. **Pre-test probability of infection should be considered for choosing between three phase bone scan and WBC scintigraphy (fractures, recent surgery, osteosynthesis, highly positive serological tests).**

**Level of evidence: 5**

P = fracture

I = bone scintigraphy or MDP or HDP

C = wbc or scintigraphy or scan or radiolabel* white blood cells or radiolabel* leucocytes or leukoscan or sulesomab or scintimun or besilesomab or BW250 or NCA90 or NCA95 or

HMPAO or oxine

O = diagnosis of infection

Search terms: (fracture) AND (bone scintigraphy or MDP or HDP) AND (wbc or scintigraphy or scan or radiolabel* white blood cells or radiolabel* leucocytes or leukoscan or sulesomab or scintimun or besilesomab or BW250 or NCA90 or NCA95 or

HMPAO or oxine) AND(diagnosis or bone or infection OR diagnostic or accuracy OR osteomyelitis)

All three together: 0 articles

1. **^18^F-FDG-PET has high diagnostic accuracy in peripheral bone infection without fracture and osteosynthesis.**

**Level of evidence: 2**

P: peripheral bone infection OR osteitis OR osteomyelitis

I: FDG-PET OR PET OR fluorodeoxyglucose OR FDG OR positron emission tomography

C: -

O: diagnosis of infection/diagnostic accuracy

Search terms: (Peripheral bone infection OR osteitis OR osteomyelitis) AND (FDG PET OR FDG-PET OR Fluorodeoxyglucose OR FDG OR positron emission tomography) AND (Diagnosis of infection OR diagnostic accuracy)

All three together: 112 articles

Included papers for thorough reading: 21 articles

Included papers after thorough reading: 15 articles

1. **Hybrid SPECT-CT WBC imaging can be performed for exact localization of infection site.**

**Level of evidence: 2**

P: peripheral bone infection OR osteitis OR osteomyelitis OR soft tissue infection

I: SPECT/CT OR hybrid imaging OR white blood cell scintigraphy

C: -

O: diagnosis of infection OR localization

Search terms: (peripheral bone infection OR osteitis OR osteomyelitis OR soft tissue infection osteomyelitis) AND (SPECT/CT OR hybrid imaging OR white blood cell scintigraphy) AND (diagnosis of infection OR localization accuracy)

All three together: 101 articles

Included papers for thorough reading: 16 articles

Included papers after thorough reading: 8 articles

1. **When having a suspicion for haematogenous spread of the infection, ^18^F-FDG-PET/CT is the first imaging modality of choice.**

**Level of evidence: 5**

P: spread of infection OR dissemination of infection OR haematogenous spread

I: FDG-PET OR FDG OR fluorodeoxyglucose OR positron emission tomography

C: -

O: diagnosis of infection

Search terms: (spread of infection OR dissemination of infection OR haematogenous spread) AND (FDG-PET OR FDG OR fluorodeoxyglucose OR positron emission tomography) AND (diagnosis of infection OR spread of infection OR dissemination)

All three together: 24 articles

Included papers for thorough reading: 1 articles

Included papers after thorough reading: 1 articles
